# Content and Validity of Claims Made about Food Parenting Practices in United Kingdom Online News Articles

**DOI:** 10.3390/ijerph19095053

**Published:** 2022-04-21

**Authors:** Chloe Patel, Lukasz Walasek, Eleni Karasouli, Caroline Meyer

**Affiliations:** 1 Warwick Manufacturting Group, Behaviour and Wellbeing Science, University of Warwick, Coventry CV4 7AL, UK; c.meyer@warwick.ac.uk; 2 Department of Psychology, University of Warwick, Coventry CV4 7AL, UK; l.walasek@warwick.ac.uk; 3 Warwick Clinical Trials Unit, Warwick Medical School, University of Warwick, Coventry CV4 7AL, UK; e.karasouli@warwick.ac.uk

**Keywords:** media, news, parents, food parenting practices, online, eating behaviours

## Abstract

The objective of this study was to qualitatively summarise the content of online news articles pertaining to food parenting practices and determine whether this content is substantiated by the scientific literature. News article data were identified and collected from United Kingdom online news published during 2010–2017 period using the News on the Web corpus. A coding framework was used to categorise the content of news articles to identify information related to food parenting practices. Then, claims made about food parenting practices were extracted from relevant news articles. Each claim was evaluated to determine the extent to which any claims were supported by the available scientific research evidence. The study identified ten claims across thirty-two relevant online news articles. Claims made across the news articles reported on the following food parenting practices: food restrictions, food-based threats and bribes, pressure to eat, use of food to control negative emotions, food availability, food preparation, and meal and snack routines. Eight out of the ten claims identified did not refer to scientific research evidence. News articles frequently lacked detail and information to explain to readers why and how the use of certain food parenting practices could have a lasting impact on children’s health outcomes. Considering the influence that news media has on parents, the reporting of food parenting practices in news articles should aim to provide a balanced view of the published scientific evidence and recognise the difficulties and barriers that prevent the use of helpful and healthy food parenting practices. The study results in this paper could be used to aid and structure of the dissemination of food parenting practice research findings in the media, inform public health education to influence perceptions of unhelpful food parenting practices, and promote parental use of responsive food parenting practices.

## 1. Introduction

Food parenting practices (FPPs) encompass a range of diverse parental approaches for influencing child eating behaviours and food consumption [1]. FPPs typically fall into one of three overarching domains: (1) coercive control; (2) structure; and (3) autonomy promotion [1]. Coercive control FPPs involve parental use of restriction, pressure to eat, food-based threats and bribes, and use of food to control negative emotions [1]. Structure-based FPPs include food rules and limits, limited/guided food choices, monitoring, meal and snack routines, modelling, food availability, food accessibility, and food preparation [1]. Finally, autonomy promotion FPPs include nutrition education, child involvement, encouragement, praise, reasoning, and negotiation [1].

A large amount of research now exists in the literature showing that FPPs can have a long-lasting effect on a child’s eating behaviour as well as on their attitudes and beliefs about food [1,2,3,4,5]. For example, coercive control FPPs such as maternal use of overt restriction is linked to potentially harmful eating behaviours such as emotional eating and eating in the absence of hunger [6,7,8,9,10]. In contrast, FPPs that are based on structure and autonomy promotion can facilitate the consumption of healthy foods (e.g., modelling of fruit and vegetable consumption) and the development of positive food-related cognitions [4,11,12].

Parents use a variety of sources to find information regarding how to feed their children, including family, friends, and media. One rich and easily accessible source of information for parents is the news media [13,14,15,16,17], with many online news outlets offering specialist webpages focused on parenting (e.g., https://www.theguardian.com/lifeandstyle/parents-and-parenting) (accessed on 12 January 2021). From 2007 on there has been an increasing trend in the UK of adults reading online news, newspapers, and magazines [18] and a concurrent decline in interest in printed news and magazines [19]. As of 2020, approximately seventy percent of adults in the UK accessed online news sources [18], in comparison to around one-third of adults who accessed printed news [19]. Indeed, with the widespread accessibility of the internet, parents now have unprecedented access to large volumes of online articles offering advice about child-rearing practices. The demand for this type of information is substantial, with health- and medical-related articles being among the most sought-after content (e.g., https://www.altmetric.com/top100/2019/) (accessed on 12 January 2021).

The mass media plays an important role in the delivery of parenting advice, and can improve parents’ knowledge, confidence, and skills [20]. Furthermore, the information conveyed in news articles can influence beliefs and social norms about FPPs, which in turn can affect how parents decide what their child should eat and how to encourage or discourage their child from consuming healthy and unhealthy foods [21,22]. Such beliefs are important according to behaviour change theories, e.g., Theory of Reasoned Action/Planned Behaviour [23], as they underpin behavioural intentions [24,25] which may subsequently lead to specific health-related behaviours [26]. Increasing parents’ knowledge and understanding of FPPs could help improve the health of children and families as well as to reduce incidence of eating disorders [27,28] and adiposity-related conditions [29,30].

At the same time, if information about FPPs in news media is lacking, inconsistent, or biased, then misconceptions and confusion might arise among parents. Indeed, this has been shown to be the case in other areas of health behaviours; medical and public health bodies have been critical of reporting on research on nutrition and eating behaviours in newspapers and Picard and Yeo stress that coverage is often based on anecdotal evidence rather than on robust empirical evidence [31]. For instance, Askelson and colleagues investigated the coverage of parenting practices related to binge drinking among college/university students, and concluded that news articles underrepresented and underplayed the role that parents can have in addressing this health issue [32]. Similarly, Kininmonth and colleagues assessed the quality of nutrition-related news articles and concluded that the public is frequently exposed to news articles containing nutrition information of poor quality about what to consume to improve one’s health [33]. In a similar vein, in the domain of sleep, Robbins and colleagues found that many popular myths about sleep that are published online are typically based on limited evidence [34]. Recent research using these myths shows that false beliefs in sleep are associated with increased engagement in behaviours that are inconsistent with recommended guidelines [35], highlighting the impact that misinformation can have on one’s health.

Existing scientific research on news media has concentrated on portrayals of child and adult obesity [36,37,38,39]. Across seven newspapers in the UK, “lack of parenting” was identified as a cause of obesity at an individual level [37]. This finding was supported by a study examining coverage in Swedish newspapers [38]. In another study, it was concluded that UK newspapers typically hold parents responsible for childhood obesity [36]. It therefore appears that media reporting may not consider the nuances of food-related parenting. This is worrying considering the significant role of FPPs in contributing to both obesity and eating-related psychopathology [1,2,40,41].

In summary, the impact that FPPs can have on child outcomes, including childhood obesity and problematic eating behaviours, makes it is important to establish how FPP-related information is portrayed by online news outlets. Furthermore, considering the importance of news media in shaping parents’ decisions, it is necessary to evaluate how the most prominent advice on online platforms relates to the growing body of empirical research in this domain. Therefore, the focus of the present study is on media representations related to FPPs. This study has two aims: first, to explore the content of online news articles pertaining to FPPs through a large dataset of online media articles, and second, to determine whether the claims made in online media articles are substantiated by the scientific literature in this field.

## 2. Methods

### 2.1. Data

Data were collected using the News on the Web (NOW) corpus (https://www.english-corpora.org/now/) (accessed on 7 April 2021) [42]. The NOW corpus is one of the largest databases of articles from magazines and newspapers published online. These data contain articles published online in the English language across twenty countries from 2010 onwards. The corpus is updated daily, and as of April 2020 contained approximately 9.75 billion words from approximately 300,000 articles [42]. For this study, we considered articles published online by UK news outlets between 2010 and July 2017, the latest date available at the time this research was conducted.

### 2.2. Article Search/Mining

We performed a string search using R [43] on each article in the corpus. Key FPP words were identified and adapted from an FPP content map that categorised FPPs under three constructs: coercive control, structure, and autonomy promotion [1]. This categorisation has been reflected in other recent systematic reviews [2,4,44]. Our objective was to identify articles that were most likely to include content related to FPPs. We therefore created two lists of words relating to FPPs (e.g., restrict, monitor, pressure) and eating (e.g., diet, family, food); see Supplementary File 1. For each article, we calculated the proportion of FPP and family words relative to the total number of words in a single article. Following visual inspection of the data by CP and LW, it was decided to further classify the data by selecting articles that were in the top tenth percentile with respect to the proportion of diet-related and FPP-related words, creating two individual datasets of news articles. We cross-referenced the two datasets to identify the articles which contained the most food-related and the most FPP-related words. As a result, our dataset contained 89 articles that contained the largest proportion of words from the two lists.

### 2.3. Analysis

The first author (CP) read all 89 identified articles to check their relevance to FPPs. Initial analysis began by reading the article title and main text and assigning FPP labels to generate raw data codes. Additionally, information on the news outlet, year of publication, tone of the article headline (negative, positive, or neutral [45]), reference to “experts”, and references to parents or grandparents were retrieved. The coding framework can be seen in Table 1.

Certain aspects of the analysis were adapted from Robbins and colleagues, who studied expert perception of sleep claims published online using the Delphi method [34]. In the present study, claims made about FPPs were extracted from news articles. A claim was defined as a statement that suggested or implied that there was a relationship between an FPP and child outcome. Any duplicate claims were consolidated into one claim. Each claim was then grouped into one of three overarching previously published FPP constructs: coercive control, structure, autonomy promotion [1]. Claims that did not fall under one of these constructs were placed under a fourth category, ‘other’. All claims were double-coded by an independent coder (MRes student with eating behaviour expertise) to determine intercoder reliability (k = 0.62) [46]. Landis and Koch recommend that intercoder values equal to or larger than 0.61 be interpreted as substantial agreement [47]. In the current study, the results were reviewed and scrutinized by all authors. Published work was used to evaluate the extent to which the content of news claims could be supported by scientific research evidence (e.g., systematic reviews and meta-analyses, randomized controlled trials (RCTs), genetic studies, and longitudinal studies).

## 3. Results

Section 3.1, Section 3.2, Section 3.3 and Section 3.4 provide a high-level description of the news articles included in the analysis. Section 3.5 onwards presents the claims identified from the included news articles.

### 3.1. News Article Characteristics

**Figure 1:**
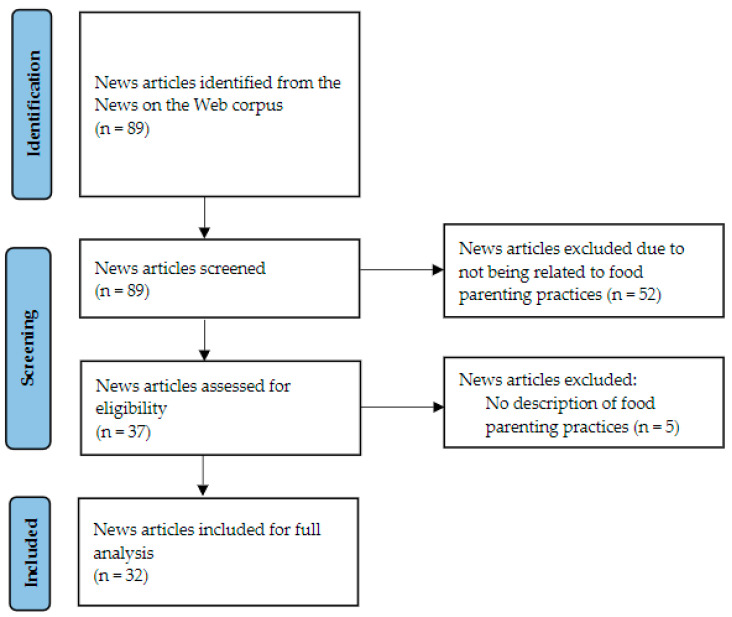
News article flow chart.

Thirty-two out of the 89 identified news articles mentioned an FPP, and an overall total of ten claims were extracted from 32 articles mentioning FPPs (Figure 1).

In keeping with previous research, articles published on a variety of newspaper website genres, such as serious (e.g., BBC News), ‘middle-market’ tabloids (e.g., Daily Mail) and tabloids (e.g., The Sun) were used [37,48] (see Table 2). Articles mentioning FPPs were most frequently published in middle-market tabloids (Daily Mail, *n* = 16). The majority of articles were written by journalists; however, two articles were personal accounts written by mothers about giving food to their children, and one article was a compilation of accounts of being given food as a child from adults who had overweight/obesity as children.

### 3.2. Headline Tone

Out of the 32 news articles, the majority (*n* = 22) had a negative or attention-seeking headline (e.g., “Are you killing your kids by feeding them too much of the wrong food?” (Mirror, 2011)), seven article headlines were considered neutral (e.g., “Do toddlers need cake as well as carrots?” (BBC News, 2010)), and three were positive (e.g., “Children won’t eat their greens? Stickers work better than ‘false’ words of praise” (Daily Mail, 2011)).

### 3.3. Expert Commentary and References

The majority of articles claimed to be referring to studies, government reports, and/or included at least one “expert” comment (study/survey/report, *n* = 2; experts, *n* = 5; study/survey/report and expert, *n* = 17). Experts cited in the articles included spokespeople from charities (*n* = 4, e.g., Caroline Walker Trust); government departments (*n* = 4, e.g., Department of Health); research or academic institutions (*n* = 10, e.g., National Centre for Social Research); healthcare professionals (*n* = 12, e.g., Dietitians); and professional bodies (*n* = 10, e.g., Royal College of Paediatrics and Child Health).

### 3.4. Parents, Mothers, Working Mothers, and Grandparents

Articles were mostly written about parents (*n* = 19). A proportion of articles specifically referred to mothers (*n* = 5), working mothers and parents (*n* = 2), and grandparents (*n* = 2). Mothers were discussed in personal accounts from individuals recalling being given food as a child in two articles. Working mothers were generally mentioned as lacking time to prepare food and meals, and grandparents were discussed as caregivers and as indulging their grandchildren with food.

### 3.5. Coercive Control

#### 3.5.1. Claim 1: Parents Restrict Food(s) from Their Children’s Diet for Health Reasons to Prevent Obesity (BBC News, 2010; Mirror, 2010; Daily Mail 2013)

Restriction was reported in three news articles in relation to parental control over their child’s weight and restricting specific food groups for health reasons. First, one news article claimed that carbohydrates are being restricted to children attending nursery in order to prevent childhood obesity (BBC News, 2010). Another news article described a mother’s account of restricting her child’s food intake to prevent the child becoming overweight (Mirror, 2010). A third news article described how a celebrity avoids giving her children pasta, bread, and rice, as she perceives these foods to be “bad” (Daily Mail, 2013).

Research evidence suggests that the use of restriction can have a detrimental impact on a child’s ability to self-regulate cues of hunger and satiety [50] and their later eating habits [1] as well as on child adiposity [51]. Although news articles used comments from experts to demonstrate this, they did not cite relevant scientific evidence. For example, longitudinal studies have identified that overt food restriction predict lower preference for fruit and vegetables and heightened preference for unhealthy, calorie-dense foods [52,53], consumption of sugar-sweetened beverages [54], emotional eating among children, increased weight status [55], and overeating [10]. However, the use of restrictive food parenting practices is a complex matter, which was not considered within the news articles. Further research evidence indicates that parental restriction is influenced by a child’s weight status, and parents are more likely to restrict food in circumstances where children have a higher body mass index (BMI) [56]. Therefore, mild use of restriction could in fact be beneficial for children with increased BMIs [57].

#### 3.5.2. Claim 2: Parents Who Pressure Their Children to Finish the Food on Their Plates Are ‘Fueling Obesity’ (Daily Mail, 2013; Daily Mail, 2015)

Pressure to eat was mentioned in two news articles. One news article discussed how pressure to eat manifests in parental insistence that their child clear their plate of food at mealtimes, which was based on the results of a population-based research study [58]. This news article provided expert commentary from the study’s author, who explained that pressuring children to eat undermines children’s internal cues of hunger and satiety.

The information presented in this news article is supported by longitudinal research indicating that parental pressure to eat more food is associated with children’s increased food consumption and eating in the absence of hunger [7,59]. This research finding is supported by a meta-analysis of FPPs and children’s eating behaviours reporting that pressure to eat is positively associated with children’s unhealthy food consumption [4]. Conversely, other studies (cross-sectional and systematic review) suggest that pressure to eat is used by parents who are concerned about their child’s weight and is in fact used in response to a lower child body mass index (BMI) [2,60,61], which was not discussed within the news articles.

The second news article was written by a mother who described pressuring her children to eat (“forcing them to eat their peas and sweetcorn”) with the intent that they consume healthy foods. This news article did not cite a research study or provide expert commentary, and research evidence suggests that although pressure to eat can be useful in achieving healthy food consumption, in the long-term it can facilitate aversions to the healthy foods children feel pressured to eat [62,63,64,65].

#### 3.5.3. Claim 3: Parents Teach Their Children Emotional Eating Behaviours (Daily Mail, 2014)

The news article mentioning the use of food to control emotions described how emotional eating is developed at a young age, often in response to stressful events. In addition, the news article advocated the notion that eating behaviours are learned from parents and that adults are more likely to eat in response to their emotions if they were provided food to control their emotions as a child.

In support of this news claim, a large twin study (*n* = 398) identified that parental use of food to control negative emotions is influential on children’s under- and overeating in response to stress [66] and that such eating behaviours are learned by children from their environment, accounting for over 70% of variance among four-year-old twins rather than genetic transmission [66,67]. This is supported by further research. First, in a large sample of mother–child dyads (*n* = 822), maternal emotional eating was found to predict the child’s emotional eating [68]. Second, another study identified that recollections of being provided food for emotional regulation as a child is strongly associated with emotional eating as an adult [69]. Third, less parental structure and lower family functioning was shown to predict emotional over- and undereating, respectively [70].

#### 3.5.4. Claim 4: Stickers Are Better Than ‘False’ Words of Praise to Encourage Children to Consume Vegetables (Daily Mail, 2011)

The use of non-food-based incentives to eat was mentioned in one news article drawing from the findings of a RCT [71]. The news article provided information and practical advice on how a non-food-based incentive to eat (i.e., giving a child stickers) can be more helpful in encouraging children to consume vegetables than parental use of verbal praise.

Among the research evidence, another RCT indicated that taste exposure and sticker rewards can increase a child’s liking for and intake of the target vegetable [72], thus supporting the news article’s claim. More recently, two systematic reviews of methods aiming to improve vegetable preference and intake, respectively, showed that vegetable consumption in young children can be increased with the use of non-food-based incentives [73,74].

### 3.6. Structure

#### 3.6.1. Claim 5: Preparing Meals from Scratch Could Decrease a Child’s Risk of Obesity (Daily Mail, 2010; Daily Mail, 2010; Daily Mail, 2012; Daily Mail, 2013; Huffington Post, 2013; The Independent, 2014; Daily Mail, 2015; Daily Mail, 2016; Daily Express, 2016; The Scotsman, 2016)

Food preparation was discussed among a number of articles (*n* = 10) and was described in a variety of contexts. The main news message related to food preparation was that parents should prepare home-cooked meals to avoid the risk of their children becoming overweight/obese.

The information presented in the news articles is consistent with the broader research literature on the topic. For example, a cross-sectional analysis of data from the UK National Diet and Nutrition Survey based on over ninety thousand eating occasions revealed that eating at home is associated with less sugar than takeaway consumption [75]. Furthermore, among a large population of adults (*n* = 11,396) more frequent home-cooked meal (≥5 times per week) consumption was shown to be associated with greater likelihood of a healthy BMI [76], and a systematic review of the determinants and outcomes of home cooking identified an association between home cooking and lower BMI [77]. Although the news article did not describe the nature of the food preparation (healthy vs. unhealthy methods, e.g., frying vs. baking), research shows that healthier food preparation is associated with a reduced risk of high weight and obesity among adolescents [78].

#### 3.6.2. Claim 6: Parents Who Take Their Children to Restaurants Are Providing Meals That Account for Approximately Half the Recommended Child Daily Calorie and Sodium Intake (The Independent, 2011)

The calorie and sodium intake of meals consumed outside of the home was discussed in one news article, and there is published research evidence to support this claim. For example, a recent analysis of *n* = 39,266 UK restaurant chain children’s meals demonstrated that they are excessively energy-dense, and contain high levels of saturated fats and salt in amounts that are inappropriate for children [79]. A longitudinal study aimed at reducing food consumption outside of the home among seven- to eleven-year-old overweight/obese children resulted in both BMI and percent body fat reductions [80]. Additionally, an analysis of *n* = 9911 meal occasions showed that children who eat at home have higher vegetable consumption, lower sweets consumption, and lower soft drink consumption [81].

#### 3.6.3. Claim 7: Children Given the Same Foods as Their Parents Are More Likely to Have Healthier Diets and Nutritious Meals (Daily Mail, 2013; The Independent, 2014)

Family meal and snack routines were mentioned within two news articles in relation to parents and children eating the same foods as well as the associated health and weight benefits.

The information presented within these news articles is largely supported by research evidence. For example, among a large sample of adolescents in a ten-year longitudinal study, family meals were found to be a protective factor against high weight and obesity [82]. Furthermore, parents who had regular family meals as adolescents reported having a healthier diet and better weight-related outcomes when compared to parents who did not experience regular family meals as adolescents [83,84]. Additionally, Dallacker and colleagues conducted a systematic review and meta-analysis of the nutritional health correlates of family meals, finding positive associations between frequent shared family meals and nutritional health among children [85].

#### 3.6.4. Claim 8: Children from Low-Income Households Are Provided an Unhealthier Diet Consisting of Takeaways or Ready Meals (Daily Mail, 2010; The Guardian, 2013; The Sun, 2017)

Food availability was mentioned in three news articles. One of these articles reported the results of a charity-commissioned report. The main message regarding food availability was the low number of fruits and vegetables consumed in low-income households, as less healthy prepared foods are more affordable.

The information reported in news articles aligned with the existing research evidence. For instance, data from a large cohort of UK adolescents (*n* = 10,736) indicate that high consumption of calorie-dense foods and low fruit and vegetable consumption is most prevalent among adolescents living in poverty (60% below the UK median household income) [86]. More recently, this association has been reported with unhealthy foods, where home availability of calorie-dense foods predicts childhood consumption of calorie-dense foods [87]. Often, research in this area is cross-sectional; however, a systematic review identified that fruit and vegetable availability in the home environment is often associated with children’s fruit and vegetable consumption [88].

### 3.7. Other

The remaining two claims (claims nine and ten) refer to aspects of FPPs, namely, weaning practices and portion size, that are not included under the FPP constructs devised by Vaughn and colleagues [1].

#### 3.7.1. Claim 9: Parents Begin Weaning Too Early Using Inappropriate Foods (Telegraph, 2011)

Early weaning was mentioned in one news article, with reference to parental provision of inappropriate foods for their child’s age and subsequent development of childhood obesity and adiposity-related conditions. Within this news article, parents’ lack of knowledge was implicated as a problem.

The NHS [89] currently advises parents to begin weaning from the age of six months by providing a variety of foods in addition to breastmilk or formula. While this information is available for parents in the public domain, it was not referred to in the news article. With regard to the suggestion of a link between early weaning and children being overweight, the research to date is unclear. One epidemiological study indicates no association between early weaning and children being overweight/obese [90]. A recent systematic review of evidence on the relationship between the introduction of complementary feeding and high weight in adolescence and adulthood concluded that study results in this research area are conflicting and ascertaining a conclusive relationship is problematic [91].

#### 3.7.2. Claim 10: Parents Overfeed Their Toddlers and Children and Provide Children with Adult-Sized Portions of Food (Daily Mail, 2010; Daily Mail, 2012; Huffington Post UK, 2013; Daily Mail, 2014; Daily Express, 2014; Daily Mail, 2016; Daily Mail, 2016; Longridge Today, 2016; The Scotsman, 2016)

Nine news articles reported information on child portion sizes and subsequent contributions to the development of high weight/obesity. These news articles reported that parents are providing portions larger than the recommended size for children. However, only one of these news articles provided advice for parents on portion sizes.

The information reported in the news articles aligns with research evidence demonstrating that consumption of large portions is associated with increased energy consumption and high child BMI [92,93]. Additionally, portion sizes determined by parents predict child BMI [94]. There are, however, several influential factors that interact with the portion sizes parents provide that were not mentioned in these articles. These include the portion sizes parents serve to themselves, parent and child BMI, perceptions of child hunger, and parents’ emotional responses, habits, and beliefs as well as children’s environmental and social influences [95].

## 4. Discussion

This study identified ten claims from thirty-two online news articles published in the UK between 2010 and 2017. The findings show that claims made by online news articles covered an array of FPPs, including those that involve coercive parental control (such as restrictions, threats and bribes, pressure to eat, and use of food to control negative emotions) and structure (such as food availability, food preparation, and meal and snack routines).

With the exception of two claims (claims two and four, relating to non-food-based incentives to eat and pressure to eat) which were based on the results of two research studies, the remaining claims did not directly refer to any published scientific research evidence. For instance, claim one was focused on the restriction of food groups from children’s diets for health reasons. Parents reading these news articles may view restricting certain foods as a simple strategy to limit their child’s intake and control their weight. What is of concern, however, is that the information presented within these articles is not supported by scientific evidence. Furthermore, the articles relating to this particular claim did not report further information to explain why restriction has a detrimental impact on child outcomes. This is true of the claim suggesting that parents teach their children emotional eating behaviours as well (claim five). This finding echoes reports that over half of nutrition-related news coverage is not based on published research, and that coverage fails to report publication journals or author names [33].

Many of the news articles analysed in the current study frequently cited just one expert opinion and/or one source of study findings, with little to no explanation around the long-term impact that FPPs can have on child outcomes. For instance, claim nine reported that parents begin weaning too early and offer age-inappropriate food to children. Yet, the news article did not provide advice to parents on the appropriate age to begin weaning, feeding strategies that could be adopted, nor on foods that could be offered to children. Similarly, no advice was offered on age-appropriate portion sizes for children in claim ten (that parents overfeed their toddlers and children and provide children with adult-sized portions of food). From the news articles analysed here, the factors affecting parental use of certain types of FPPs were rarely reported and described. This is important to acknowledge, as parental motivation for use of certain FPPs is complex [1]. For instance, claim nine reports that children from low-income households are provided an unhealthier diet comprised of takeaways or convenience meals. However, lower parental cooking self-efficacy is a factor that is associated with fewer meals made from basic or raw ingredients [96]. Furthermore, parental stress is another factor that has been shown to influence the likelihood of serving a homemade meal in the home as well as the use of coercive FPPs [97]. Claim ten reports that parents overfeed their toddlers. Indeed, the literature demonstrates that large portion sizes are associated with increased energy consumption and child BMI [92,93]. However, research evidence reports that parents provide portion sizes they themselves have learned to be appropriate for their child [98]. As might be expected, the news articles fail to convey the nuance and context-dependence of FPP use.

The results of this study further demonstrate that a large amount of research evidence is disregarded in the reporting of FPPs. There was a lack of direct references to academic literature within news articles. This is concerning, as lack of detailed reporting on FPPs could lead to continued parental engagement with FPPs that are known to have poorer outcomes on children’s health despite their use by parents being well-intentioned (e.g., use of food to control negative emotions) [1]. Indeed, previous sleep myth-based research shows that presentation of misinformation influences continued engagement in behaviours that are not supported by recommended guidelines [35], although further research is needed to confirm such findings in relation to FPPs.

Despite this, we found that certain claims made in the online news articles could be supported by scientific evidence. This stands in contrast to previous research findings on beliefs about sleep (*n* = 20), where reports were found to have a largely ambiguous evidence base [34]. There was one claim identified in the current study, claim nine, where the research evidence connecting early weaning and childhood obesity remains unclear [91].

From the news articles included in the study, the findings highlight rare news media reporting of FPPs involving structure and those that aim to support child autonomy, such as parental role modelling of healthy food consumption, monitoring, nutrition education, and child involvement, all of which are shown to have positive, healthy child outcomes [1,4]. Reviews of the literature in this area indicate that modelling healthy food consumption, providing a healthy home food environment, and FPPs that support encouragement and independence in children’s eating behaviours are associated with a healthier dietary intake and eating behaviours [1,4,85]. Academics, researchers, and press offices in these settings who engage with news media outlets could aid future reporting by considering the readership prior to discussions. The research community could promote the use of structure- and autonomy-promoting FPPs and highlight the benefits of these practices when appropriate.

From the news articles analysed, there was a lack of reporting on fathers. The reason for this is likely due to the role that mothers have on child feeding from the postpartum period onwards. Research reports that mothers have increased responsibility for family work, including feeding their children, when compared to fathers [99]. This finding is in line with the FPP research field generally, although the inclusion of fathers in FPP-related research is improving [100]. Previous research reports that many news articles are reported in a ‘negative’ tone [38,46]. Although our findings cannot be directly compared, most of the news articles analysed in the current study presented a negative tone and attention-seeking headlines.

The news media have a platform to shape social norms and beliefs, and therefore may influence parents’ understanding of FPPs. In order to counteract these practices, the British Nutrition Foundation charity created the “Previous facts behind the headlines” (https://archive.nutrition.org.uk/nutritioninthenews/headlines.html) (accessed on 8 December 2021) website with the aim of providing an evidence-based summary of health research that is published in the news. The National Health Service provides a similar website, “Behind the Headlines” (https://www.nhs.uk/news/), (accessed on 8 December 2021) in an effort to clarify health news in the media. There is an opportunity for the media to aid public health efforts to encourage parents to provide recommended portion sizes using, for instance, the British Nutrition Foundation’s information on healthy living. Given the implications of poor reporting of health issues in the media (potentially due to the lack of details within news articles, lack of input from researchers/experts/authorities, shortening or removal of important information and details at editorial level, and the need to ensure that a news article is appealing or attention-worthy to the reader), the results of this study could be used to aid the development of guidelines for health behaviour FPP-related reporting for journalists.

The results of the present study raise possibilities for further study recommendations. First, research could investigate correlations between news content, parental knowledge, and behaviour. Second, there may be merit in exploring alternative media sources such as social media-based online discussions, which may be more frequently accessed by parents. Third, it would be interesting to understand whether the content of news articles related to FPPs affects parental thinking around FPPs and the types of foods offered to their children. Finally, in claim three of the results, that parents teach their children emotional eating behaviours, it was reported in the articles that eating behaviours are transmitted from parent to child. Since there is research evidence supporting this [66,68,101], there may be value in extended research exploring the links between parents’ recollections of being provided food as children and this influence on their use of food-related parenting practices with their own children.

The study results are somewhat limited by the nature of the data. The news articles and the claims extracted are written in lay language and can be subject to multiple interpretations. To alleviate this issue, each extracted claim was coded by a second reviewer against each news article in order to counteract bias. One issue is that our articles, which date as far back as 2010, may include views on FFPs that are now outdated. Although the relatively small sample size did not allow us to explore time trends, we believe that the articles’ age is of little importance in the context of online content. Each of the news articles covered in the present paper can be easily discovered and accessed by searching for FPP-related content online. As such, it is likely that these articles are being consulted by parents who seeking information about the optimal ways of feeding their children. Finally, another potential limitation is that our analysis focused on UK-based publications only. However, the fact that the articles appear online means that they can be accessed by anyone around the world who speaks English. Nonetheless, future research may focus on cross-cultural differences in the coverage of FPPs in online news.

## 5. Conclusions

This research was deemed necessary due to the importance of the news media as a source of health and medical information for parents [102]. The current study determined that a large amount of research evidence was disregarded in news media articles. What is concerning, and a missed opportunity for the news media, is the lack of detail and information provided within news articles to explain why and how the use of some FPPs can have a long-lasting and sometimes unintentional impact on a child’s relationship with food, BMI, and other health outcomes. Future portrayal of FPPs in news articles should acknowledge the difficulties and barriers that prevent the use of helpful and healthy FPPs and include practical strategies for overcoming barriers such as fussy or selective eating. While it is not the news media’s responsibility to provide health advice, it is important that researchers and practitioners are informed about what is published in the news around FPPs, as there may be an opportunity for interventions to address myths or parental misperceptions due to what they have read. The reporting on FPPs in news media should aim to provide a balanced view of the published scientific evidence. This is important because of the news media’s role as a powerful source of influence on social norms, beliefs, and health issues, to the extent that the media can even act as part of the health provider community [103].

## Figures and Tables

**Table 1:** Coding framework for news articles.

Variable	Description
Month, Year	Month and year of article’s publication
News outlet	Name of the online news platform
Central topic	The article’s central topic of discussion, e.g., childhood obesity
Headline tone	Positive/negative/neutral/no headline
Reference(s)	The article refers to findings from a research study/survey, poll or book release
Article voice	Who is the article written by? e.g., Journalist, personal account.
Articles focus	Does the article focus on mothers, fathers, parents, caregivers, or other family members?
Expert commentary	The article cites comments from an expert, e.g., professional body, dietitian.
Advice	The article provides advice for the reader and/or wider society
Food-related parenting practice	The article mentions a food-related parenting practice, e.g., restriction, pressure to eat, using food to control negative emotions, modelling.

**Table 2:** Articles (*n* = 32) by newspaper genre and publication title.

Genre	Publication Title	*n* Articles	Average Weekly Usage as of 2017 (%) ^1^
Serious	The Independent	2	6
	BBC News	2	47
	The Guardian	1	14
	The Telegraph	1	6
	The Yorkshire Post	1	10 *
		7	
Middle-market tabloid	Daily Mail	16	14
	Daily Express	2	NR
	Huffington Post UK	1	14
	The Scotsman	1	NR
		20	
Tabloid	Mirror	3	6
	The Sun	1	5
		4	
Local	Longridge Today	1	10 *
Total		32	

^1^ Out of *n* = 2112 surveyed; * survey question referred to ‘website of local paper’ [49].

## Data Availability

Restrictions apply to the availability of these data. Data was obtained from [https://www.english-corpora.org/now/, accessed on 28 February 2022] and are available [https://www.english-corpora.org/now/, accessed on 28 February 2022] with the permission of [https://www.corpusdata.org/purchase.asp, accessed on 28 February 2022].

## Supplementary Materials

The following supporting information can be downloaded at: https://www.mdpi.com/article/10.3390/ijerph19095053/s1; Table S1: Corpus search terms.
